# Prevalence and Risk Factors for Malignant Nodal Involvement in Early Esophago-Gastric Adenocarcinoma: Results From the Multicenter Retrospective Congress Study (endosCopic resectiON, esophaGectomy or Gastrectomy for Early Esophagogastric Cancers)

**DOI:** 10.1097/SLA.0000000000006496

**Published:** 2024-09-02

**Authors:** Philip H. Pucher, Saqib A. Rahman, Pradeep Bhandari, Natalie Blencowe, Swathikan Chidambaram, Tom Crosby, Richard P.T. Evans, Ewen A. Griffiths, Sivesh K. Kamarajah, Sheraz R. Markar, Nigel Trudgill, Timothy J. Underwood, James A. Gossage, on behalf of the CONGRESS collaborative

**Affiliations:** * Department of General Surgery, Portsmouth University Hospitals NHS Trust, Portsmouth, UK; † Imperial College London, London, UK; ‡ University of Bristol, Bristol, UK; § Velindre University NHS Trust, Cardiff, UK; ∥ Department of Upper GI Surgery, Queen Elizabeth Hospital, University Hospitals Birmingham NHS Foundation Trust, Birmingham, UK; ¶ Institute of Immunology and Immunotherapy, University of Birmingham, Birmingham, UK; # Oxford University NHS Foundation Trust, Oxford, UK; ** University Hospitals Southampton NHS Foundation Trust, Southampton, UK; †† Guy’s and St Thomas’ Hospital NHS Foundation Trust, London, UK

**Keywords:** esophageal cancer, endoscopic resection, early cancer

## Abstract

**Objective:**

The aim of this study was to quantify lymph node metastasis (LNM) risk and outcomes following treatment of early esophago-gastric (EG) adenocarcinoma.

**Background:**

The standard of care for early T1N0 EG cancer is endoscopic resection (ER). Radical surgical resection is recommended for patients perceived to be at risk of LNM. Current models to select organ-preserving versus surgical treatment are inconsistent.

**Methods:**

CONGRESS is a UK-based multicenter retrospective cohort study. Patients diagnosed with clinical or pathological T1N0 EG adenocarcinoma from 2015 to 2022 were included. Outcomes and rates of LNM were assessed. Cox regression was performed to assess the impact of prognostic and treatment factors on overall survival.

**Results:**

A total of 1601 patients from 26 centers were included, with median follow-up 32 months (IQR 14–53). 1285/1612 (80.3%) underwent ER, 497/1601 (31.0%) underwent surgery. Overall rate of LNM was 13.5%. On ER staging, tumour depth (T1bsm2-3 17.6% vs T1a 7.1%), lymphovascular invasion (17.2% vs 12.6%), or signet cells (28.6% vs 13.0%) were associated with LNM. In multivariable regression analysis, these were not significantly associated with LNM rates or survival. Adjusting for demographic and tumour variables, surgery after ER was associated with significant survival benefit, HR 0.33 (0.15–0.77), *P*=0.010.

**Conclusions:**

This large multicenter data set suggests that early EG adenocarcinoma is associated with significant risk of LNM. These data are representative of current real clinical practice with ER-based staging, and suggests previously held beliefs regarding reliability of predictive factors for LNM may need to be reconsidered. Further research to identify patients who may benefit from organ-preserving versus surgical treatment is urgently required.

Whereas surgical resection was once the mainstay of treatment for management of early (T1N0) esophago-gastric (EG) adenocarcinoma, endoscopic resection (ER) is now the accepted standard and in many cases is thought to offer curative organ-preserving treatment. Radical surgery is associated with major morbidity risk^1^ and deleterious effect on quality of life.^2,3^ It is generally reserved for tumors with high-risk features which are believed to place patients at high risk of lymph node metastasis (LNM), which cannot be addressed through ER.^4–6^ Such features are generally considered to include the presence of lymphovascular invasion (LVI) or submucosal invasion to a depth of >500 μm (T1b sm2+); some guidelines additionally include poorly differentiated tumors.^4,5^

Counseling patients on their potential LNM risk, and whether to pursue surgical resection, is challenging. There is some disagreement between international guidelines on what risk factors must be considered.^4–6^ Furthermore, estimation of the theoretical risk of LNM conferred by these risk factors is based almost exclusively on historical analyses of small case series of predominantly surgical specimens, consisting typically of 50 to 200 patients.^7–10^ Estimating LNM risk for ER specimens, using published data from surgical specimens, is problematic due to differences in the way that ER and surgical specimens are prepared and variability in pathological assessment, with potential understaging in surgical specimens.^10,11^ Available data are thus highly heterogenous, with reported LNM rates for T1N0 disease ranging from <2% for T1a disease^10^ to as high as 50% or more in higher risk tumors.^12,13^

A large-scale granular data set is urgently needed to better understand risks, management, and outcomes of early EG cancer, and guide patient counseling and joint decision-making. The aim of this study was to quantify LNM risk and outcomes following treatment of early EG adenocarcinoma with curative intent in a large multicenter data set.

## METHODS

### Study Design and Setting

CONGRESS (endosCopic resectiON, esophaGectomy or gastrectomy foR Early esophagogastric cancerS) was conducted as a multicenter retrospective cohort study with a structure modeled on, and methodology developed in partnership with members of, previous international research collaboratives.^1,14,15^ A database capturing diagnostic, demographic, treatment, and outcome variables was designed and piloted by a multidisciplinary steering group that included surgeons, oncologists, gastroenterologists, and methodologists. This database was transcribed to an online platform for anonymised data submission (Research Electronic Data Capture, REDCap).

Open invitations to participate were circulated via specialist societies, social media, and personal communication with a predominant focus on UK centers; EG cancer management in England and Wales is centralized to 35 tertiary hospitals. In addition to UK centers, 1 Swedish center (Karolinska Institutet) also took part. Each center’s local lead was responsible for ensuring their own relevant ethical permissions and study registration to comply with local protocols.

### Inclusion Criteria

Patients diagnosed 2015 to 2022 (inclusive) were eligible for inclusion, with the follow-up period extending until the database closure date in July 2023.

The aim was to capture outcomes for all patients who received treatment with curative intent for T1N0 cancer, based on available staging. To capture real-world outcomes for T1N0 disease, the allowed diagnostic criteria were pragmatic and included any patients with cT1N0 undergoing curative therapy, as well as patients undergoing surgery or ER for high-grade dysplasia (HGD) with subsequent pT1N0—as these would also be subject to the same decision-making regarding subsequent surveillance or surgery. Only patients with a histological diagnosis of adenocarcinoma, or initially columnar dysplasia, were included in this analysis. Patients who received palliative or no treatment for any reason were excluded.

### Data Capture

Demographic data included age, gender, and comorbidities. Initial diagnostic data and up to 3 treatment rounds were captured to account for patients who may have had initial ER followed by additional treatment (including repeat ER, surgery, or oncological therapy). Clinical and survival outcomes were recorded.

### Statistical Analysis

Patient who underwent surgery were compared with those that did not using appropriate nonparametric statistical tests (χ^2^ and Kruskal-Wallis). Predictive factors for LNM were compared with final surgical specimen pathology.

To assess differences between LNM risk for ER and surgical specimen-based staging within the same patient group, we compared histopathological findings, along with corresponding LNM risk, for the patients who underwent ER followed by surgery. Where the surgical specimen contained no residual primary tumor, endoscopic pathological results were used.

Multivariable regression was performed to assess the feasibility of a predictive model for LNM risk following ER based on demographic, clinical and pathological variables. Factors affecting overall survival were assessed using multivariable Cox regression analysis. Missing data were handled by multiple imputation by chained equations. *P*<0.05 was considered statistically significant. STROBE guidelines were adhered to in reporting of results (Supplemental Data Appendix 1, Supplemental Digital Content 1, http://links.lww.com/SLA/F289).

## RESULTS

A total of 1841 patients from 26 centers were included. Median follow-up was 32 months (IQR 14–53). Further analysis was confined to patients with confirmed adenocarcinoma or columnar type high-grade dysplasia (HGD), giving a cohort size of 1601 (1197 adenocarcinoma, 404 HGD). Data collection was good, with low rates of data missingness: <1% in initial staging and surgical outcome data. Missingness of endoscopic resection pathology data was <1% for tumor depth, differentiation, and presence of signet cells, 11% for LVI.

**FIGURE 1:**
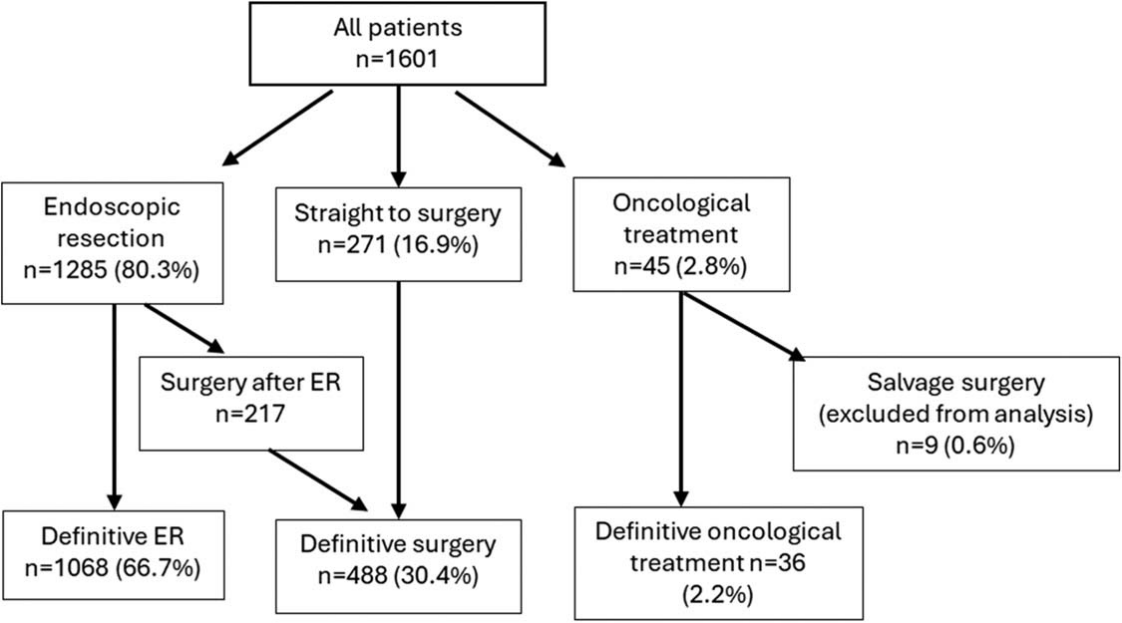
Flowchart of patients meeting inclusion criteria and treatment pathway. ER indicates endoscopic resection.

Initial clinical staging (Table 1) for these patients was T1 in 978 (61.1%), TX for 348 (21.8%) and T0 or HGD in 274 (17.1%). Initial staging investigations performed were variable and more common in patients with confirmed adenocarcinoma, and included CT scan (73%), positron emission tomography (30.5%), endoscopic ultrasound (31.5%), and staging laparoscopy (6.5%). Initial management of these patients was predominantly endoscopic resection (1285/1601, 80.3%), of which 217/1285 (16.9%) of patients went on to have surgery based on high-risk features or patient preference (Fig. 1). A total of 271/1601 (16.9%) of patients were primarily managed with surgery. Where a reason was given, most patients who went straight to surgery were either deemed not endoscopically resectable (170/270 valid responses, 62.9%), or in a small number of cases underwent primary surgery due to patient choice (22/270, 8.1%). Ultimately, 497 patients (31.0% of all patients) with clinically early disease at presentation underwent radical surgery.

**TABLE 1:** Initial cT Stage Before Index Treatment

T stage	n %
T0 (ie, high-grade dysplasia)	274 (17.1)
T1 (ie, T1a/T1b not reported)	390 (24.4)
T1a	375 (23.4)
T1b	213 (13.4)
Tx (ie, cancer not visible on imaging, early cancer without explicit T stage)	348 (21.8)

### Patient Demographics

Median patient age was 71 years. Patients were predominantly male, with distal esophageal tumors (Table 2). When comparing patients undergoing surgery versus those who did not, surgical patients were more likely to be younger (68 vs 72 y, *P*<0.001), have a Charlson comorbidity score of 0 (59.2% vs 46.5%, *P*<0.001), and had more advanced tumors, with a greater proportion of surgical patients demonstrating poorly differentiated tumor cells (23.3% vs 8.5%, *P*<0.001), present signet cells (8.9% vs 2.1%, *P*<0.001), or less favorable cT stage (T1b 22.3% vs 9.1%, *P*<0.001).

**TABLE 2:** Patient Demographics

	Overall	No surgery	Surgery	*P*
n	1612	1115	497	
Age at diagnosis	71.00 [64.00, 77.00]	72.00 [65.00, 78.00]	68.00 [60.00, 74.00]	<0.001
Patient sex	—	—	—	0.798
Male	1272 (78.9)	879 (78.8)	393 (79.1)	—
Female	339 (21.0)	235 (21.1)	104 (20.9)	—
Missing	1 ( 0.1)	1 ( 0.1)	0 ( 0.0)	—
Charlson score 0	812 (50.4)	518 (46.5)	294 (59.2)	<0.001
Site of pathology	—	—	—	<0.001
Proximal eosphagus	9 ( 0.6)	5 ( 0.4)	4 ( 0.8)	—
Middle esophagus	104 ( 6.5)	76 ( 6.8)	28 ( 5.6)	—
Distal esophagus	870 (54.0)	639 (57.3)	231 (46.5)	—
Gastro-esophageal Junction	383 (23.8)	290 (26.0)	93 (18.7)	—
Stomach—cardia/body	126 ( 7.8)	62 ( 5.6)	64 (12.9)	—
Stomach — distal/antrum/pylorus	120 ( 7.4)	43 ( 3.9)	77 (15.5)	—
Pathology within Barrett	1136 (70.5)	851 (76.3)	285 (57.3)	<0.001
Histology on Biopsy				
High-grade dysplasia	404 (25.1)	331 (29.7)	73 (14.7)	<0.001
Adenocarcinoma	1208 (74.9)	784 (70.3)	424 (85.3)	
Extent of tumor differentiation	—	—	—	<0.001
Well	319 (19.8)	252 (22.6)	67 (13.5)	—
Moderate	536 (33.3)	362 (32.5)	174 (35.0)	—
Poor	211 (13.1)	95 ( 8.5)	116 (23.3)	—
HGD	474 (29.4)	379 (34.0)	95 (19.1)	—
Missing	72 ( 4.5)	27 ( 2.4)	45 ( 9.1)	—
Presence of Signet cells				<0.001
No	1538 (95.4)	1086 (97.4)	452 (90.9)	
Yes	67 ( 4.2)	23 ( 2.1)	44 ( 8.9)	
Missing	7 ( 0.4)	6 ( 0.5)	1 ( 0.2)	
Initial tumor T stage	—	—	—	<0.001
T0 / HGD	274 (17.0)	235 (21.1)	39 ( 7.8)	—
T1 (subgroup not reported)	390 (24.2)	210 (18.8)	180 (36.2)	—
T1a	375 (23.3)	308 (27.6)	67 (13.5)	—
T1b	213 (13.2)	102 ( 9.1)	111 (22.3)	—
Tx	348 (21.6)	251 (22.5)	97 (19.5)	—
Missing	12 ( 0.7)	9 ( 0.8)	3 ( 0.6)	—

### Procedural Outcomes

Following endoscopic resection, no complications were reported in 1198/1285 (93.2%) of cases. The most common reported complications included bleeding in 42/1285 (3.3%) and perforation in 11/1285 (0.9%) of cases.

Considering patient outcomes after radical surgery, where outcome data were available, complications occurred in 284/453 patients (62.7%), which were of Clavien-Dindo grade 1 or 2 in 168 patients (37.0%), grade 3a in 41 (9.1%), 3b in 28 (6.1%), 4 in 38 (8.4%). In-hospital mortality was 2.0% (9 patients). The median length of stay was 10 days (IQR 7–17.75), and a median of 22 lymph nodes were harvested in each case (IQR 15–31).

### Predictors of Lymph Node Metastasis

The overall rate of surgical specimen LNM was 67/497 (13.5%). As expected, more advanced nodal stage corresponded to worse survival (*P*=0.006, Supplemental Data Appendix 2, Supplemental Digital Content 1, http://links.lww.com/SLA/F289). Assessing histopathology of all surgical specimens, where recorded, LVI was present in 85/473 cases (17.9%). Tumor cell differentiation was poor in 107/459 (31.8%). Signet ring cells were present in 47/494 (9.5%).

**FIGURE 2:**
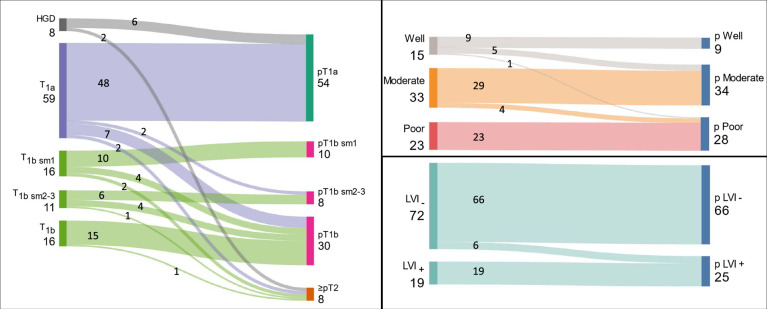
Flowchart illustrating differences between ER and final (surgical) pathology for patients undergoing initial ER followed by surgery. Individual plots for T stage, worst tumor differentiation grade, and LVI. Patients with initial R1 ER margins excluded from this analysis.

When comparing ER-based and subsequent surgical pathological staging variables for the subset of patients who underwent surgery after ER, there was significant discordance between endoscopic and surgical staging. Of the patients who underwent ER followed by surgery, 110 patients had R0 resection with complete pathological data, of which 40 (36%) had subsequent surgical specimen pathology exhibiting discordant T stage or LVI status (Fig. 2). The overall rate of LNM for this group was 13.8% (30/217). The rate of LNM across varying T stages was T1a=9.8%, T1b sm1=14.8%, T1b sm2-3=17.9%. Significant rates of LNM were seen in patients without any reported ER pathological risk factors (ie, patients without any of the following risk factors: positive deep or circumferential ER margins, poor differentiation, or T1b sm2 or greater depth of invasion), 8/52 (15.3%). Comparing rates of N+ between ER and surgical specimens (Table 3), analyses of surgical specimens returned less advanced tumors (lesser T stage, lower incidence of poorly differentiated tumors or LVI) (*P*<0.001 for all comparisons), but higher rates of LNM, suggesting overall potential understaging in surgical specimens.

**TABLE 3:** Comparison of Surgical and ER Pathology With Subsequent LNM Status for Patients Undergoing Surgery After ER. WHERE no Residual Cancer was Seen in the Surgical Specimen, Final Primary Tumor Staging Was Based on ER Specimen

	Surgical staging			ER staging		
	N0	N+	%N+	N0	N+	%N+
T stage						
T0	0	0	NA	7	1	12.5
T1a	73	4	5.2	79	6	7.1
T1b (not otherwise reported)	64	13	16.9	45	10	18.2
T1b sm1 (≤500 um)	17	1	5.6	25	5	16.7
T1b sm2-3 (>500 um)	22	2	8.3	28	6	17.6
T2 or greater	9	10	52.6	1	0	0.0
Worst differentiation grade						
Well	32	3	8.6	28	6	17.6
Moderate	77	10	11.5	75	10	11.8
Poor	55	14	20.3	53	9	14.5
Presence of LVI						
Yes	52	16	23.5	48	10	17.2
No	121	13	9.7	111	16	12.6
Presence of signet cells						
Yes	8	3	27.3	5	2	28.6
No	178	27	13.2	180	27	13.0

**FIGURE 3:**
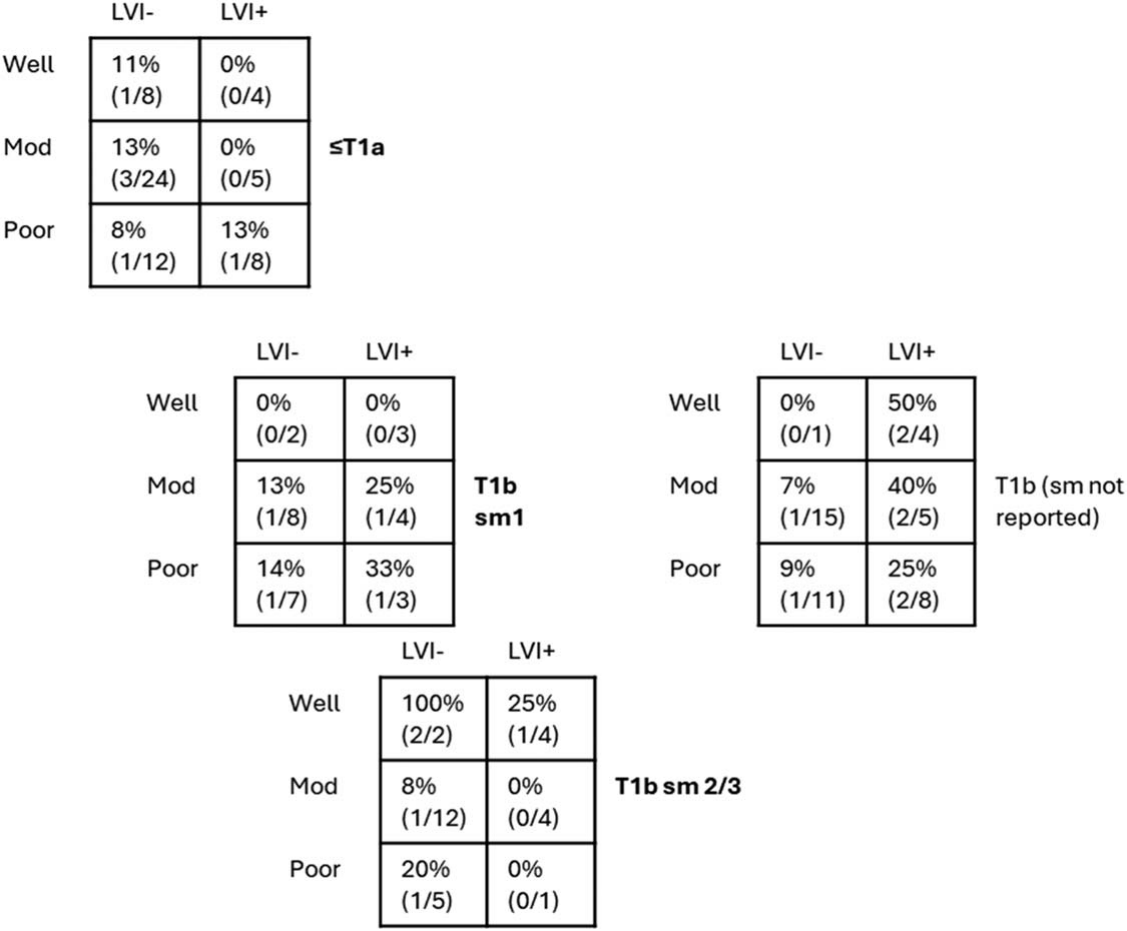
Observed nodal metastasis risk in patients undergoing surgery after endoscopic resection. Rates of nodal metastasis in surgical specimen, stratified by T stage, differentiation, and lymphovascular invasion seen on endoscopic resection specimen. Percentages with absolute numbers in parentheses. Numbers do not equate to total surgical volume as patients not included here if any missing histological data or initial pathology showed dysplasia only.

Cross tabulated by ER-derived LVI, differentiation, and T stage, the number of patients for each subgroup were low; surgical specimens positive for LNM were, however, seen across almost all groups (Fig. 3).

Multivariable regression analysis to derive a prognostic model for LNM based on histological and demographic variables resulted in a statistically nonsignificant model with poor calibration (Supplemental Data Appendix 3, Supplemental Digital Content 1, http://links.lww.com/SLA/F289).

### Cox Regression Analysis for Overall Survival

For all patients, after adjusting for age, sex, Charlson comorbidity score, tumor site, histological subtype, differentiation, Barrett, presence of signet cells and cT stage before treatment (Supplemental Data Appendix 4, Supplemental Digital Content 1, http://links.lww.com/SLA/F289), age (HR 1.07, 95% CI: 1.05–1.09, *P*<0.001) and Charlson score 0 (0.57 (0.44–0.74), *P*<0.001) were significantly associated with survival. In terms treatment variables, surgery was not significantly associated with survival advantage for unselected patients (HR 0.72, 95% CI: 0.50–1.03, *P*=0.070).

Analyzing only patients who underwent ER, after adjusting for demographic and disease variables (Table 4), a significant survival benefit was seen for patients undergoing surgery [HR 0.33 (0.15–0.77), *P*=0.010], with poorer survival for older patients [1.08 (1.05–1.011), *P*<0.001 and 0.001] and positive ER circumferential margin [2.51 (1.47–4.29), *P*=0.001].

**TABLE 4:** Multivariable Cox Regression for Overall Survival in Patients Undergoing Endoscopic Resection as Primary Treatment (n=1118)

Variable	HR (95% CI)	*P*
Age	1.08 (1.05–1.11)	**<0.001**
Sex		
Male	Reference	—
Female	0.91 (0.52–1.58)	0.726
Charlson score=0	0.75 (0.46–1.23)	0.253
Barrett esophagus	1.61 (0.85–3.06)	0.142
Site		
Proximal esophagus	Reference	—
Middle esophagus	2.86 (0.32–25.79)	0.350
Distal esophagus	2.49 (0.32–19.09)	0.380
GOJ	2.3 (0.27–19.23)	0.443
Stomach (cardia/body)	2.3 (0.2–25.91)	0.501
Stomach (distal/antrum)	7.14 (0.69–73.73)	0.099
Worst endoscopic pT		
Dysplasia	Reference	—
pT1a	4961669.71 (0–Inf)	0.996
pT1bSM1	7956213.13 (0–Inf)	0.996
pT1bSM2-3	3472217.36 (0–Inf)	0.996
Worst endoscopic differentiation		
Good	Reference	—
Moderate	1.27 (0.69–2.36)	0.442
Poor	1.23 (0.56–2.66)	0.608
Signet ring +	1.43 (0.65–3.15)	0.375
LVI +	0.63 (0.13–3.05)	0.566
Deep margin +	1.99 (1.08–3.67)	**0.027**
Circumferential margin +	2.51 (1.47–4.29)	**0.001**
Surgery after ER	0.33 (0.15–0.77)	**0.010**

## DISCUSSION

CONGRESS represents the largest known granular data set (containing detailed demographic, disease, and outcome data) for early EG cancer to date, presenting contemporary management strategies and real-world outcomes for 26 centers. The overall rate of LNM of 13.5% is higher than has been reported in previous series, with one pooled analysis suggesting rates of 4% for T1a and 23% for T1b disease.^16^ Previously reported histological risk factors such as LVI, T stage, and cell differentiation grade did not exhibit clear association when comparing ER staging to LNM risk or overall survival. In multivariable Cox regression analysis, surgery was associated with a strong survival benefit after primary ER.

In modern clinical practice, the need to decide between either organ-preserving or surgical therapy is predominantly informed by ER-based pathological staging. Predictive models for LNM should therefore be based upon ER specimen pathology, rather than surgical pathology, if they are to be valid. Published prediction models for LNM in T1b cancer exemplify the limitations of existing reports in that such studies are often based on surgical specimens (rather than ER), low numbers, and outdated practice: Lee et al^17^ reported a risk prediction system for LNM in T1 disease based upon 258 surgical specimens from 5 institutions over 11 years (2000–2011), whereas Gotink et al^18^ included 248 patients treated predominantly with primary surgery between 1989 and 2016. These data should therefore not be used to predict LNM based on ER. ER specimens are assessed differently (with typically smaller slices prepared for analysis and therefore closer scrutiny); surgical specimens after ER may also contain multifocal or residual disease with different final staging. The known variability in the assessment of surgical specimens^19^ is thought to further contribute to potential understaging of disease if considering surgical specimens alone.

In the present data set, despite large patient numbers, we did not find a significant association between pathological variables and LNM risk. This is a substantial finding which calls into question the validity of preoperative counseling of patients based on initial histopathological data. It also calls into question the validity of previous risk assessment studies, many of which were based upon surgical pathology rather than ER. It may be that the increasingly recognized heterogeneous nature of EG cancer, and interaction of multiple risk factors, means that it is difficult or impossible to accurately predict this from endoscopic specimens. It is equally possible, however, that discordance in this this real-world multicenter data set instead reflects the known variability in staging workup discordant reporting between pathologists.^19–22^ The variability of staging investigations also suggests that improvements in the standardization of workup of these patients may be required, which could improve the pretreatment detection of nodal metastases, though some reports have highlighted that CT and positron emission tomography-CT may be of limited sensitivity or utility in HGD or early cancer.^23^ Differences in specimen preparation and pathologist variation may mean that relevant risk factors may be more or less likely to be identified on pathological assessment. Current UK guidelines recommend joint assessment of specimens by 2 pathologists, one of who should be a gastrointestinal specialist, for Barrett’s dysplasia only;^24^ data on the number and specialist interest of pathologists involved in the CONGRESS specimens was not collected. The number of T1a tumors which went forward to surgery also reflects the importance of other nonpathological considerations which might influence the need for surgery, such an endoscopic appearances or multifocal disease, which were not recorded in this data set. Other factors such as variable time intervals (and potentially resulting tumor progression) between ER and surgery may further complicate pathology-based risk assessment. These issues highlight the need for detailed and prospective study to better understand factors associated with LNM risk. Without a standardized, reliable assessment of specimens, it may not be possible to adequately assess individual patient prognosis based on ER alone.

This inability to identify a significant pattern of multifactorial adjusted risk factors, both for LNM in regression analysis as well as overall survival in Cox regression, in the CONGRESS data set, further complicates the existing dilemma of how to counsel patients with clinically staged early EG cancer. First, over 10% of all patients with tumors that were endoscopically staged T1b sm1 or less, traditionally thought to be the lowest risk group, were found to have LNM at surgery, without any significant association to LVI or tumor differentiation status. Should all patients with early EG cancer, regardless of stage, be offered surgical consultation? Second, what is an acceptable risk for LNM? The American Gastroenterological Association alludes to a “minimal chance” of LN or distant metastasis as representing <2% and that a perceived risk above this should be considered for surgery.^5^ Justification of such a threshold is sometimes given as surgery being considered a viable option if the risk of LNM outweighs the risk of mortality after esophagectomy. However, this discounts the significant consequences of undergoing surgery, such as at least temporarily decreased quality of life, and does not consider the fact that patients may accept differing degrees of risk. Some patients may desire maximal risk reduction, or to avoid the recurrent interventions and potential anxiety associated with surveillance, and be more likely to request surgery. In contrast, other patients may wish instead to maximize their quality of life with organ-preserving treatment (ER), and may accept a potentially discounted life expectancy in return for avoiding surgery even in higher-risk tumors.^25,26^ There remains also a question about the potential efficacy of adjuvant therapies such as chemotherapy, radiotherapy, or brachytherapy, which could potentially further contribute to disease control after ER in early EG cancer.

The findings of this study, that surgery is significantly associated with improved overall survival in early EG cancer after ER, is at odds with some other published findings. Tankel et al^27^ reported equivalent survival for surveillance and esophagectomy after ER of high risk T1b esophageal cancer; however, this was in a small patient group, with only 27 patients in the observation group over a study period of 11 years (2012–2022). Kamarajah et al^28^ reported a large US (National Cancer Data Base, NCDB) analysis of patients with T1a and T1b esophageal cancer; ER had equivalent long-term survival compared with primary surgery after propensity score matching for demographic and disease variables. However, the database does not account for differences in ER and surgical staging. Surgical specimens were therefore potentially understaged compared to ER, and represented in fact more advanced disease (and thus benefitted from esophagectomy). An analysis by the same group of gastric cancer data found that ER was inferior to surgery for gastric cancers.^29^

Data for CONGRESS were entered through a collaborative group model based on previously successful studies with many of the same personnel;^1,15^ levels of data missingness were low and surgical outcome data entered into the CONGRESS database closely mirror those reported in the compulsory UK national EG cancer database (NOGCA),^30^ strongly supporting the internal validity of this data set. In multivariable Cox regression analysis, CONGRESS data have suggested that surgery after ER is associated with improved overall survival—however, significant differences between groups and an absence of cause of death or recurrence data mean that some of this difference may result from incomplete adjustment within the regression model, highlighting the need for prospective study. Furthermore, the lack of data on disease-related mortality or recurrence type (local, nodal, or systemic), limits some of the conclusions that can be drawn. The relatively high incidence of LNM even in ER-staged low risk groups is surprising, and suggests the possible presence of additional risk factors not captured here or addressed in current guidelines. Despite the large numbers of included patients, cross tabulated analysis of histological subgroups also resulted in small numbers for each group when assessing for LNM risk which suggests a sample size limitation for statistical analysis. The real-world and contemporaneous nature of these data, however, strongly supports the generalizability of the reported findings to daily clinical decision-making in current practice. Novel treatments such as sentinel lymph node biopsy^31^ may in future offer an alternative to radical surgery, but are not yet proven.

Patients with early EG cancer, along with their clinicians, face a dilemma when it comes to deciding on the optimal treatment modality. Based upon this large predominantly UK-based data set, the risk of LNM appears greater, and less predictable in current practice, than previously reported. Many of these findings are discordant with currently accepted evidence, suggesting an urgent need for re-evaluation of staging, treatment, and quality control processes. These data should be used to inform joint decision-making, and highlights the need for urgent prospective study.

## CONGRESS COLLABORATIVE CITABLE CO-AUTHORS

Tarig Abdelrahman, Khalid Akbari, Leo Alexandre, Hasan Ali, Bilal Alkhafaff, Anuradaha Alwis, Antonios Athanasiou, Evan Best, Khalid Bhatti, Nick Bird, Alex Boddy, Matt Bonomaully, Amir Botros, Leo Brown, Benjamin Byrne, Richard Byrom, Beatriz Carrasco Aguilera, David Chan, Carissa Choh, Hollie Clements, Peter Coe, Lauren Crocker, Andrea Cross, Vinutha DayaShetty, Niell Dempster, Alexander Dermanis, Massimiliano Di Pietro, Simon Dwerryhouse, Ahmed Elshaer, Nada Elzahed, Sarah Epton, Matthew Forshaw, Nana Gael, Lewis Gall, Ismael Ghazzi, Leeying Giet, Hasan Haboubi, George Hanna, Paul Healy, Jonathan Hoare, Sung Hong, Faisal Ibrahim, Anchal Jain, Chenchen Ji, Courtney Johnson, Sharib Khan, Frederik Klevebro, Bhaskar Kumar, Jie Li, Steven Lindley, Anantha Madhavan, Ash Mahendran, Henrik Maltzmann, Michel Martin, Sotiris Mastoridis, Euan McLaughlin, David Mitton, Krishna Moorthy, Magnus Nilsson, Robert O’Neill, Mervyn Owusu-Ayim, Sally Pan, Simon Parsons, Pradeep Patel, Ian Penman, Abeera Pervez, Chris Peters, Shaun Preston, Oliver Priest, Tom Ritchie, Ioannis Sarantitis, Negar Sharafi, Katie Siggens, Aayush Sinha, Richard Skipwowrth, Naim Slim, Maria Soupashi, Sophie Stephens, Jennifer Straatman, Jav Sultan, Cheuk-Bong Tang, Nav Thavanesan, Mie Thu, Paul Turner, Bhamini Vadhwana, Ravi Vohra, Shajahan Wahed, Michael White, Thomas Whittaker, Vincent Wong, Susannah Woodrow, Sebastian Zeki.

## Supplementary Material
