# Supplementary material for: Prevalence and Risk Factors for Malignant Nodal Involvement in Early Esophago-Gastric Adenocarcinoma: Results From the Multicenter Retrospective Congress Study (endosCopic resectiON, esophaGectomy or Gastrectomy for Early Esophagogastric Cancers)
Source: Ann Surg. 2024 Sep 2;281(3):363–70. doi: 10.1097/SLA.0000000000006496 (PMC11809703; doi:10.1097/SLA.0000000000006496)
Supplement: Supplementary file 1 [file sla-281-363-s001.docx]

**SUPPLEMENTAL DATA**

**APPENDIX 1:** STROBE Statement—Checklist of items that should be included in reports of ***cross-sectional studies***

|  | Item No | Recommendation | Page No |
| --- | --- | --- | --- |
| **Title and abstract** | 1 | (*a*) Indicate the study’s design with a commonly used term in the title or the abstract | 1 |
|  |  | (*b*) Provide in the abstract an informative and balanced summary of what was done and what was found | 3 |
| Introduction | | | |
| Background/rationale | 2 | Explain the scientific background and rationale for the investigation being reported | 3 |
| Objectives | 3 | State specific objectives, including any prespecified hypotheses | 3 |
| Methods | | | |
| Study design | 4 | Present key elements of study design early in the paper | 4 |
| Setting | 5 | Describe the setting, locations, and relevant dates, including periods of recruitment, exposure, follow-up, and data collection | 4 |
| Participants | 6 | (*a*) Give the eligibility criteria, and the sources and methods of selection of participants | 4 |
| Variables | 7 | Clearly define all outcomes, exposures, predictors, potential confounders, and effect modifiers. Give diagnostic criteria, if applicable | 4 |
| Data sources/ measurement | 8* | For each variable of interest, give sources of data and details of methods of assessment (measurement). Describe comparability of assessment methods if there is more than one group | *5* |
| Bias | 9 | Describe any efforts to address potential sources of bias | n/a |
| Study size | 10 | Explain how the study size was arrived at | n/a |
| Quantitative variables | 11 | Explain how quantitative variables were handled in the analyses. If applicable, describe which groupings were chosen and why | 5 |
| Statistical methods | 12 | (*a*) Describe all statistical methods, including those used to control for confounding | 6 |
|  |  | (*b*) Describe any methods used to examine subgroups and interactions | 6 |
|  |  | (*c*) Explain how missing data were addressed | 6 |
|  |  | (*d*) If applicable, describe analytical methods taking account of sampling strategy | 6 |
|  |  | (*e*) Describe any sensitivity analyses | n/a |
| Results | | | |
| Participants | 13* | (a) Report numbers of individuals at each stage of study—eg numbers potentially eligible, examined for eligibility, confirmed eligible, included in the study, completing follow-up, and analysed | 6 |
|  |  | (b) Give reasons for non-participation at each stage | n/a |
|  |  | (c) Consider use of a flow diagram | Fig1 |
| Descriptive data | 14* | (a) Give characteristics of study participants (eg demographic, clinical, social) and information on exposures and potential confounders | 6 |
|  |  | (b) Indicate number of participants with missing data for each variable of interest | Tab2 |
| Outcome data | 15* | Report numbers of outcome events or summary measures | 7 |
| Main results | 16 | (*a*) Give unadjusted estimates and, if applicable, confounder-adjusted estimates and their precision (eg, 95% confidence interval). Make clear which confounders were adjusted for and why they were included | 7 |
|  |  | (*b*) Report category boundaries when continuous variables were categorized |  |
|  |  | (*c*) If relevant, consider translating estimates of relative risk into absolute risk for a meaningful time period | n/a |
| Other analyses | 17 | Report other analyses done—eg analyses of subgroups and interactions, and sensitivity analyses | 7 |
| Discussion | | | |
| Key results | 18 | Summarise key results with reference to study objectives | 8 |
| Limitations | 19 | Discuss limitations of the study, taking into account sources of potential bias or imprecision. Discuss both direction and magnitude of any potential bias | 10 |
| Interpretation | 20 | Give a cautious overall interpretation of results considering objectives, limitations, multiplicity of analyses, results from similar studies, and other relevant evidence | 10 |
| Generalisability | 21 | Discuss the generalisability (external validity) of the study results | 10 |
| Other information | | | |
| Funding | 22 | Give the source of funding and the role of the funders for the present study and, if applicable, for the original study on which the present article is based | n/a |

*Give information separately for exposed and unexposed groups.

**Note:** An Explanation and Elaboration article discusses each checklist item and gives methodological background and published examples of transparent reporting. The STROBE checklist is best used in conjunction with this article (freely available on the Web sites of PLoS Medicine at http://www.plosmedicine.org/, Annals of Internal Medicine at http://www.annals.org/, and Epidemiology at http://www.epidem.com/). Information on the STROBE Initiative is available at www.strobe-statement.org.

| **Appendix 2.** Kaplan-Meier survival curve stratified by surgical nodal stage |
| --- |
|  |

**Appendix 3: Multivariable model to predict nodal metastasis**

A logistic regression model was then generated to assess for the predictive ability of variables available before surgery and after endoscopic resection for the risk of lymph node metastasis. We excluded patients who were staged pre-surgery as having T2 or higher disease, and those who were labelled as having T1b disease without subdividing this into SM1/2/3, leaving a total of 165 patients for whom sufficient data was available. Characteristics of this cohort are shown below.

|  | N0 | N1+ | p |
| --- | --- | --- | --- |
| n | 147 | 18 |  |
| agebin (%) |  |  | 0.396 |
| <60 | 39 (26.5) | 7 (38.9) |  |
| 60-69 | 56 (38.1) | 8 (44.4) |  |
| 70-79 | 45 (30.6) | 3 (16.7) |  |
| 80+ | 7 (4.8) | 0 (0.0) |  |
| sex = female (%) | 38 (25.9) | 2 (11.1) | 0.278 |
| charlsonbin (%) |  |  | 0.9 |
| 0 | 88 (59.9) | 11 (61.1) |  |
| 1 | 37 (25.2) | 5 (27.8) |  |
| ≥2 | 22 (15.0) | 2 (11.1) |  |
| site (%) |  |  | 0.955 |
| Prox/Mid Oesophagus | 15 (10.2) | 2 (11.1) |  |
| Distal oesophagus | 90 (61.2) | 12 (66.7) |  |
| Gastro-oesophageal junction | 20 (13.6) | 2 (11.1) |  |
| Stomach | 22 (15.0) | 2 (11.1) |  |
| histo_fin (%) |  |  | 0.594 |
| Adenocarcinoma | 95 (64.6) | 13 (72.2) |  |
| HGD | 45 (30.6) | 5 (27.8) |  |
| Squamous cell carcinoma | 7 (4.8) | 0 (0.0) |  |
| barretts = Yes (%) | 104 (70.7) | 16 (88.9) | 0.177 |
| Endoscopic p Stage |  |  | 0.187 |
| No residual/dysplasia only | 10 (6.8) | 2 (11.1) |  |
| T1a | 81 (55.1) | 5 (27.8) |  |
| T1bSM1 | 26 (17.7) | 5 (27.8) |  |
| T1bSM23 | 30 (20.4) | 6 (33.3) |  |
| Differentiation |  |  | 0.871 |
| Well | 26 (17.7) | 4 (22.2) |  |
| Mod | 62 (42.2) | 7 (38.9) |  |
| Poor | 34 (23.1) | 5 (27.8) |  |
| Missing | 25 (17.0) | 2 (11.1) |  |
| LVI |  |  | 0.64 |
| No | 94 (63.9) | 13 (72.2) |  |
| Yes | 34 (23.1) | 4 (22.2) |  |
| Missing | 19 (12.9) | 1 (5.6) |  |
| Signet Ring status |  |  | 0.621 |
| No | 143 (97.3) | 17 (94.4) |  |
| Yes | 3 (2.0) | 1 (5.6) |  |
| Missing | 1 (0.7) | 0 (0.0) |  |
| Deep Margin |  |  | 0.306 |
| Not involved | 70 (47.6) | 12 (66.7) |  |
| Involved | 61 (41.5) | 5 (27.8) |  |
| Missing | 16 (10.9) | 1 (5.6) |  |
| Circumferential Margin |  |  | 0.055 |
| Not involved | 71 (48.3) | 14 (77.8) |  |
| Involved | 44 (29.9) | 3 (16.7) |  |
| Missing | 32 (21.8) | 1 (5.6) |  |
| Lymph node involvement | 0 (0.0) | 18 (100.0) |  |

Missing data was handled using a K-nearest neighbour approach, with K=5. Model predictive performance was assessed by bootstrapping, with 1000 bootstrap resampled datasets used and performance averaged across these. The above covariates were unable to reliably predict lymph node positivity in patients undergoing surgical resection (c-index 0.520). Similarly calibration of the model demonstrated no relationship between predicted and observed probability of involvement.

**
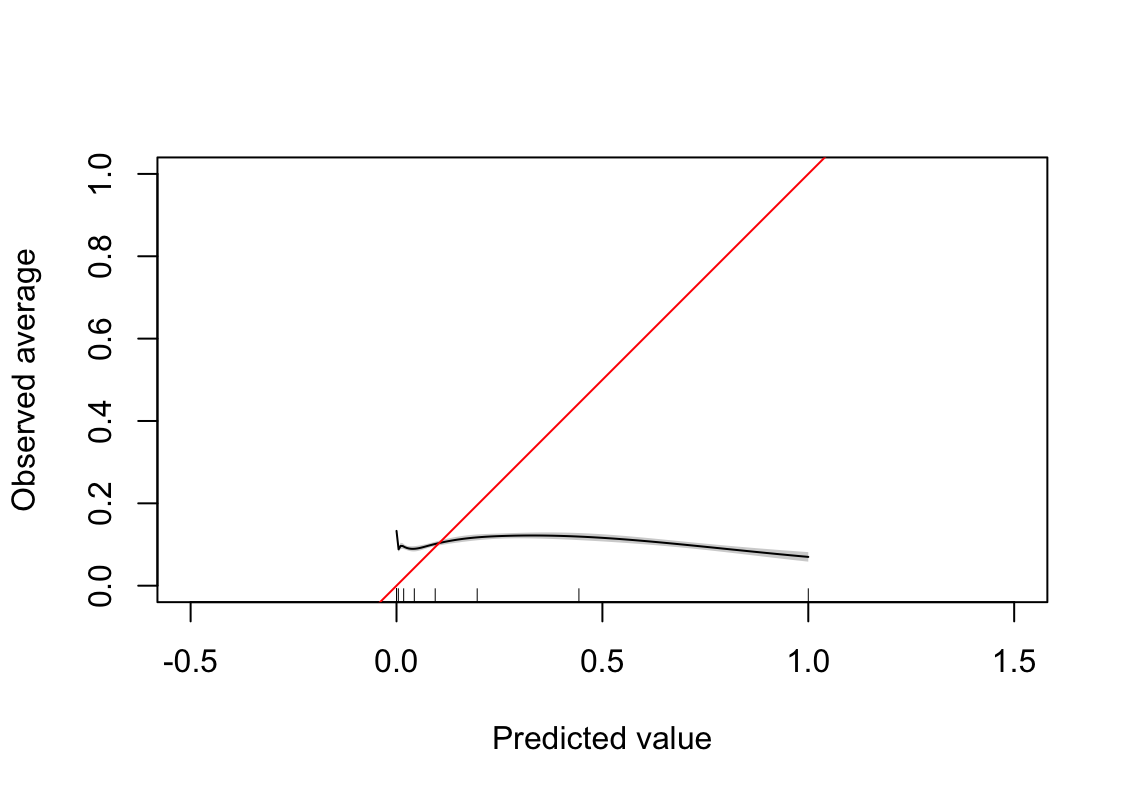
**

**Appendix 4: Multivariable predictive model for survival**

To further quantify the impact of covariates on survival, univariate and multivariate hazard ratios were then calculated. Missing data was handled by multiple imputation by chained equations.

|  |  | Univariate | | Multivariate | |
| --- | --- | --- | --- | --- | --- |
|  |  | HR (LCI-UCI) | Pvalue | HR (LCI-UCI) | Pvalue |
| Age | | 1.07 (1.05-1.08) | **0.000** | 1.06 (1.04-1.07) | **0.000** |
| Female Sex | | 0.9 (0.68-1.19) | 0.465 | 0.84 (0.63-1.12) | 0.226 |
| Charlson score = 0 | | 0.54 (0.42-0.69) | **0.000** | 0.64 (0.5-0.82) | **0.000** |
| Site | Proximal oesophagus |  |  | Ref |  |
|  | Middle oesophagus | 1.16 (0.35-3.83) | 0.817 | 1.03 (0.31-3.42) | 0.971 |
|  | Distal oesophagus | 1.14 (0.36-3.57) | 0.836 | 1.19 (0.37-3.8) | 0.786 |
|  | GOJ | 1.29 (0.41-4.1) | 0.680 | 1.34 (0.41-4.37) | 0.637 |
|  | Stomach (cardia/body) | 1.92 (0.58-6.36) | 0.291 | 1.39 (0.4-4.81) | 0.616 |
|  | Stomach (distal/antrum/plyorus | 1.81 (0.55-5.98) | 0.336 | 1.59 (0.46-5.53) | 0.472 |
| Histology | Adenocarcinoma |  |  | Ref |  |
|  | HGD | 0.69 (0.5-0.94) | **0.020** | 0.84 (0.49-1.45) | 0.542 |
|  | other | 1.69 (0.89-3.21) | **0.107** | 2.19 (1.1-4.32) | **0.024** |
|  | SCC | 1.51 (1.01-2.23) | **0.042** | 1.67 (0.99-2.81) | **0.054** |
| Barretts |  | 0.59 (0.46-0.74) | **0.000** | 0.78 (0.55-1.09) | 0.140 |
| Differentiation | Well | 0.95 (0.68-1.31) | 0.747 | Ref |  |
|  | Moderate |  |  | 1.07 (0.76-1.5) | 0.725 |
|  | Poor | 1.3 (0.88-1.9) | 0.184 | 1.46 (0.98-2.17) | 0.062 |
|  | Anaplastic | 0 (0-Inf) | 0.991 | 0 (0-Inf) | 0.992 |
|  | HGD | 0.72 (0.5-1.02) | 0.064 | 0.9 (0.54-1.51) | 0.702 |
| Signet Cells |  | 0.8 (0.41-1.57) | 0.534 | 0.55 (0.27-1.12) | 0.097 |
| cT Stage | T0/HGD | Ref |  | Ref |  |
|  | T1 (i.e. T1a/T1b not reported) | 1.53 (1.02-2.31) | **0.041** | 1.17 (0.69-1.99) | 0.564 |
|  | T1a | 1.25 (0.81-1.95) | 0.318 | 0.95 (0.54-1.68) | 0.874 |
|  | T1b | 1.8 (1.13-2.87) | **0.013** | 1.33 (0.74-2.39) | 0.338 |
|  | Tx | 1.74 (1.16-2.63) | **0.008** | 1.19 (0.71-2) | 0.523 |
| Underwent Surgery | | 0.59 (0.45-0.78) | **0.000** | 0.70 (0.52-0.96) | **0.028** |
